# The detection and verification of carbapenemases using ertapenem and Matrix Assisted Laser Desorption Ionization-Time of Flight

**DOI:** 10.1186/1471-2180-14-89

**Published:** 2014-04-10

**Authors:** Åsa Johansson, Josefine Ekelöf, Christian G Giske, Martin Sundqvist

**Affiliations:** 1 Department of Clinical Microbiology, Växjö, Sweden; 2 School of Natural Science, Linnaeus University, Kalmar, Sweden; 3 Clinical microbiology, Karolinska Institutet – MTC, Karolinska University Hospital, Stockholm, Sweden; 4 Department of Laboratory Medicine, Clinical Microbiology, Örebro University Hospital, Örebro, Sweden

**Keywords:** MALDI-TOF, Carbapenemases, Klebsiella pneumoniae, Pseudomonas aeruginosa, Detection, Ertapenem

## Abstract

**Background:**

The increase in carbapenemase producing Enterobacteriaceae and *Pseudomonas aeruginosa* is a significant threat to modern medicine. A rapid detection of carbapenemase production in *Klebsiella pneumoniae* and *Pseudomonas aeruginosa* is of importance for the institution of correct antibiotic treatment and infection control measures.

**Results:**

Standardised inoculums of *K. pneumoniae* or *P. aeruginosa* were incubated at 37°C with ertapenem in 15 and 120 min followed by centrifugation. The supernatant was applied on a steel target plate, covered with HCCA matrix and analysed using a Microflex^TM^ (Bruker Daltonics) in the mass range of 4–600 Da. The assay detected and separated KPC from other carbapenemases in *K. pneumoniae* after only 15 min incubation. In *P. aeruginosa*, however, only 8/14 isolates of VIM-producing *P. aeruginosa* were detected. None of the tested carbapenemase negative isolates displayed a pattern of hydrolysis of ertapenem.

**Conclusions:**

This assay allows for a very rapid detection and verification of KPC (45 min including the preparation steps) and MBL production (150 min) in *K. pneumoniae* and can be performed using standard matrix. However, the study revealed the need for optimization of the substrate/species combination in assays for the detection of carbapenemases in *P. aeruginosa* using MALDI-TOF.

## Background

The increase in carbapenemase-producing Enterobacteriaceae and *Pseudomonas aeruginosa* is a significant threat to modern medicine [1]. As treatment options are very limited, infection control measures are important to contain carbapenemase-producing isolates in health care settings. Rapid detection of carbapenemase-producers is a decisive for adequate infection control measures to be undertaken. The methods used so far for the detection of carbapenemases have been phenotypic methods or PCR [2,3] Recently, Matrix Assisted Laser Desorption Ionization-Time Of Flight (MALDI-TOF) has been introduced in clinical microbiology for species identification and during the last two years a few studies have shown the proof of concept regarding the detection of β-lactamases using this technology [4-6]. These studies have either analyzed a small set of strains [4] or focused on the detection of hydrolysis rather than the verification of specific enzymes [5-8]. All studies have used different protocols and different sets of species/enzyme combinations. In the present study we present a method for the simultaneous detection and discrimination of KPC from the metallo-β-lactamases (MBL) NDM and VIM in *Klebsiella pneumoniae* and the possibility of verification of VIM in *Pseudomonas aeruginosa* through a time dependent hydrolysis assay and the addition of specific inhibitors, APBA (3-aminophenylboronic acid) and DPA (2.6-Pyridinecarboxylic acid).

## Results

### Stability of ertapenem

Ertapenem was stable after one week and six months when stored at −20°C, but degraded after one week when stored at +4°C. The frozen aliquots were used for further analysis.

### Klebsiella pneumoniae (n = 40)

**Figure 1:**
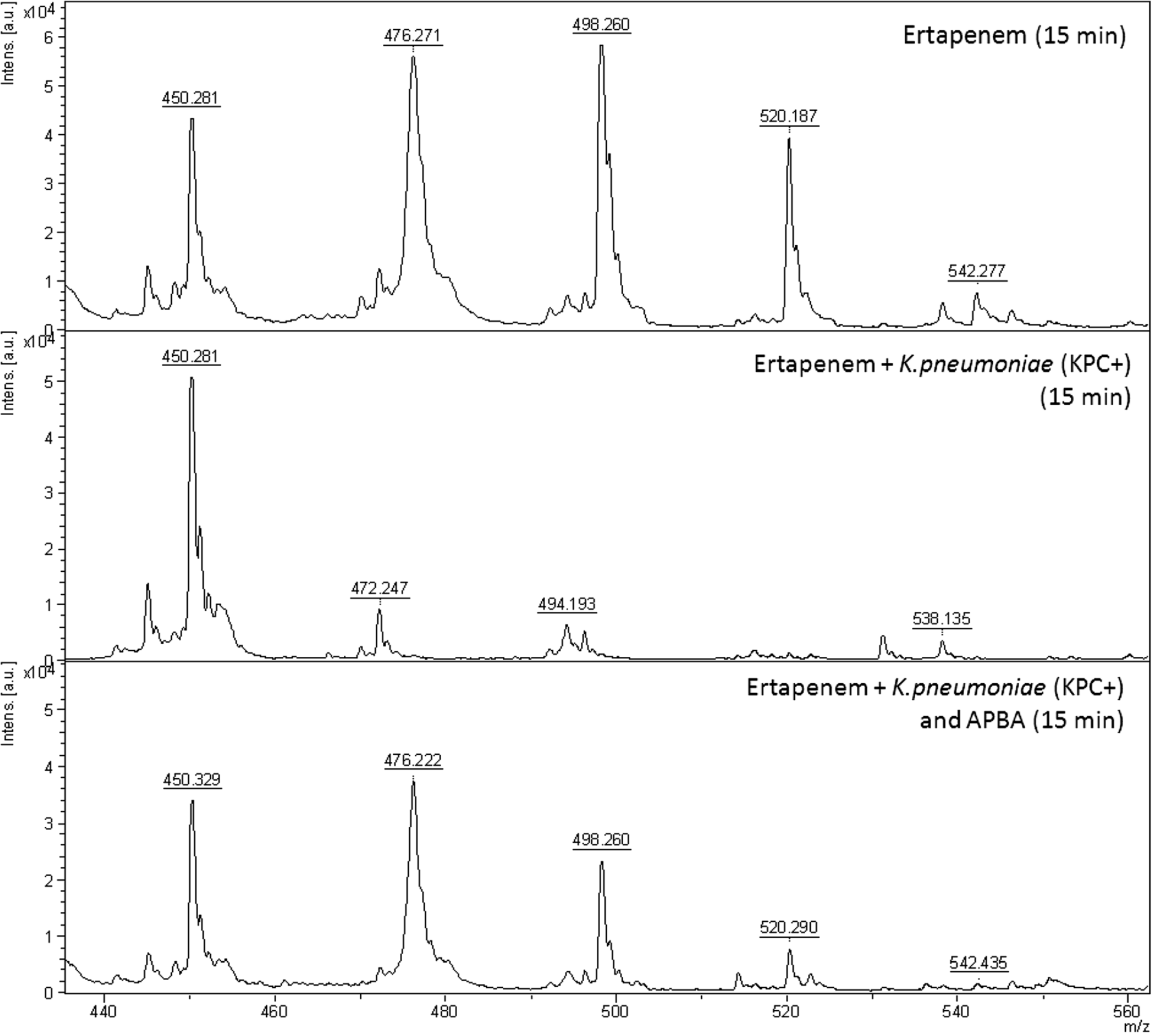
**Mass spectrum showing the non hydrolysed pattern of ertapenem (top), the full hydrolysis of ertapenem of a KPC producing *K. pneumoniae *** **after 15 min (middle) and the effect of the supplement of APBA inhibiting the KPC mediated hydrolysis of ertapenem (bottom).**

**Figure 2:**
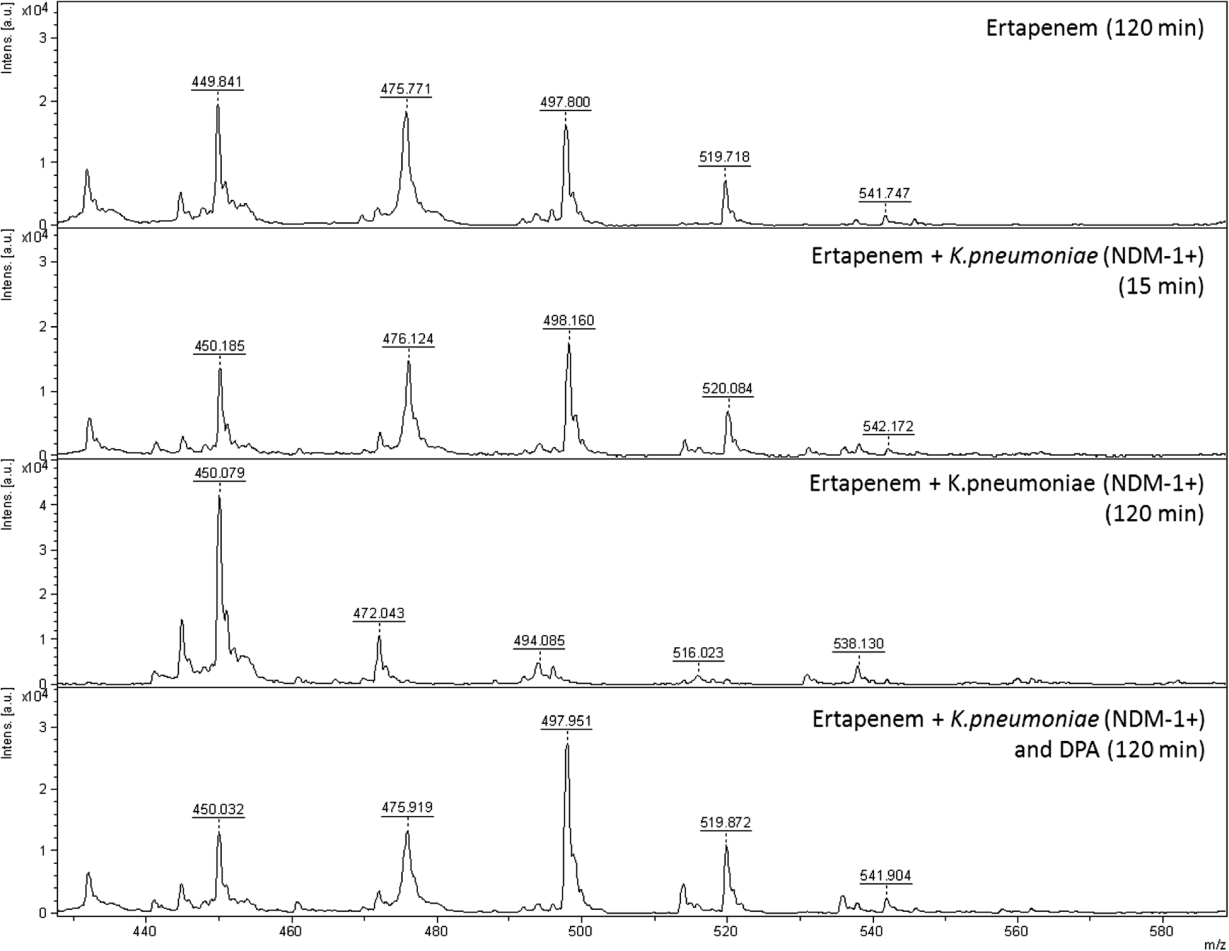
**Mass spectrum showing the non hydrolysed pattern of ertapenem (top), The non hydrolysed pattern of ertapenem after 15 min incubation together with NDM producing *K. pneumoniae *** **(middle top), the full hydrolysis of ertapenem of a NDM-producing *K. pneumoniae *** **after 120 min (middle bottom) and the effect of the supplement of DPA inhibiting the NDM mediated hydrolysis of ertapenem (bottom).**

**Figure 3:**
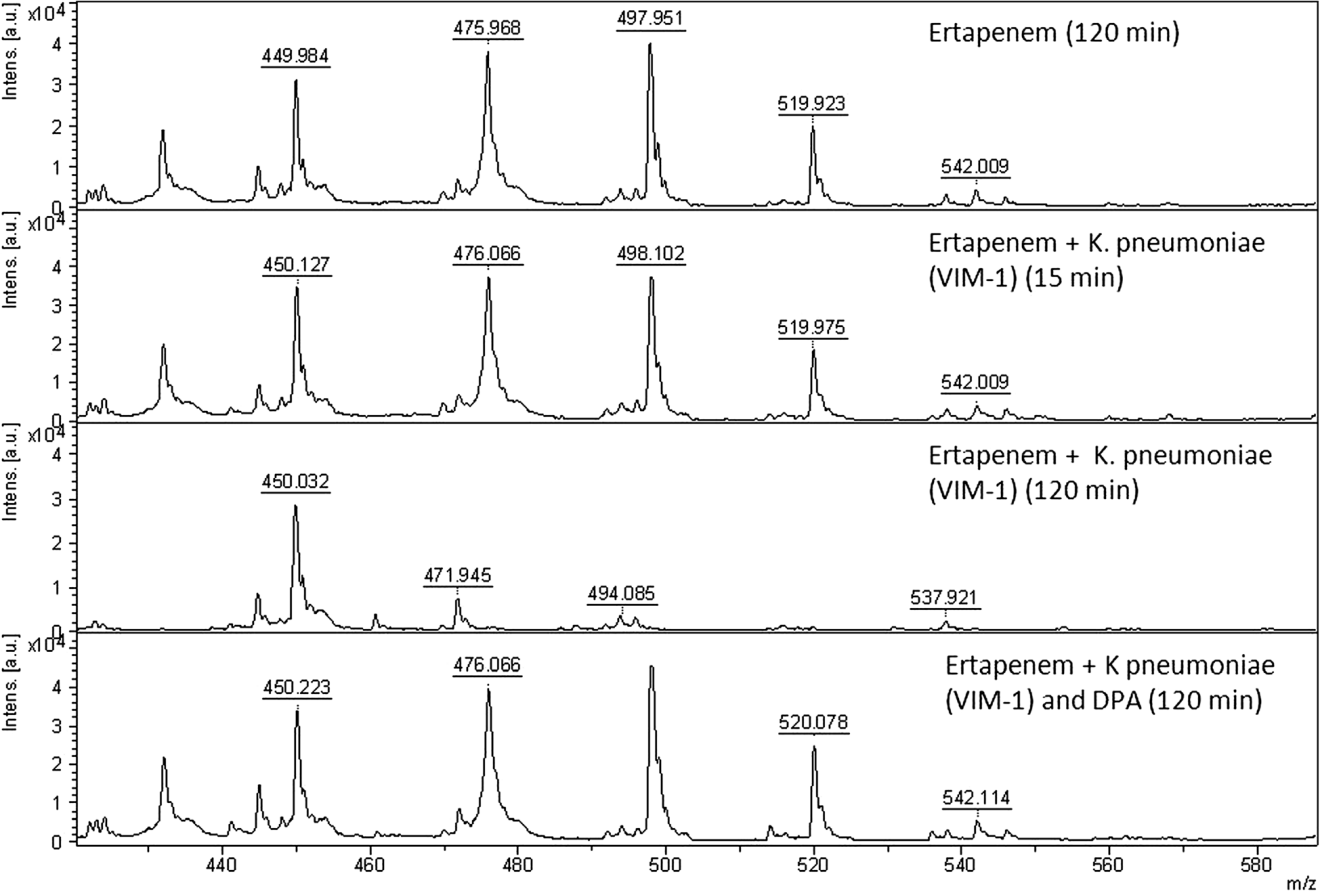
**Mass spectrum showing the non hydrolysed pattern of ertapenem (top), The non hydrolysed pattern of ertapenem after 15 min incubation together with VIM producing *K. pneumoniae *** **(middle top), the full hydrolysis of ertapenem of a VIM-producing *K. pneumoniae *** **after 120 min (middle bottom) and the effect of the supplement of DPA inhibiting the VIM mediated hydrolysis of ertapenem (bottom).**

All the KPC producing *K. pneumoniae* (n = 10) displayed the specific ertapenem hydrolysis peak pattern after 15 min incubation (Figure 1, middle). As no potassium was included in this assay only the sodium ions of hydrolysed ertapenem with the m/z ratios of 450.5, 472.5, 494.5, 516.5 and 538.5 were detected. The hydrolysis was inhibited by APBA (Figure 1, bottom) as only the unhydrolysed forms of ertapenem were detected with the m/z ratios of 476.5, 498.5, 520.5 and 542.5. The NDM- (n = 4) and VIM-producing (n = 3) *K. pneumoniae* isolates did not hydrolyse ertapenem in 15 minutes but hydrolysis was observed after 120 minutes incubation (Figures 2 and 3). The hydrolysis of VIM- and NDM-enzymes was fully inhibited by DPA (Figures 2 and 3). At these concentrations the inhibition was 100% specific for the respective enzyme. Ertapenem was not hydrolysed by the ATCC 13882 or by the clinical isolates with classical ESBL or acquired AmpC (n = 12) (Table 1). All *K. pneumoniae* (n = 11) in the validation panel with KPC, NDM, or VIM enzymes were correctly assigned as KPC- or MBL-producers while none of the isolates with OXA-48 enzyme (n = 3) displayed hydrolysis after 2 h while all showed the pattern of ertapenem hydrolysis after 24 h. A summary of the results is presented in Table 1.

**Table 1:** A synthesis of the results showing the basic data in relation to hydrolysis

	**Species**	**Mechanism (n)**	**Hydrolysis, n, time**	**Meropenem MIC (mg/L)**	**Imipenem MIC (mg/L)**	**Ertapenem MIC (mg/L)**
**Test panel**	***K. pneumoniae***	KPC-2 (4)		4 - >32	4 - >32	2 - >32
		KPC-3 (2)	10/10			
		KPC (4)	15 min			
		VIM-1 (3)	3/3	>32	32 - >32	8 - >32
			120 min			
		NDM-1 (4)	4/4	>32	>32	>32
			120 min			
		Classic ESBL (6)	0/6	na	na	0.016 - 0.125
			120 min			
		Acquired AmpC 6)	0/6	0.064 - 0.125	0.064 - 0.25	0.032 - 2
			120 min			
	***P. aeruginosa***	VIM-1 (2)		>32	>32	>32
		VIM-2 (6)	6/10			
		VIM (2)	120 min			
		IMP-14 (1)				
		Carba R	0/10	8 - >32	4 - >32	>32
		(non-MBL) (10)	120 min			
**Validation panel**	***A. baumannii***	OXA 23-like (n = 2)	4/4	>32	>32	>32
		OXA 24-like (n = 1)	24 h			
		OXA 58-like (n = 1)				
	***P. aeruginosa***	VIM-1 (3)	2/4	>32	>32	>32
		VIM-2 (1)	120 min			
	***K. pneumoniae***	OXA-48 (3)	3/3 24 h	4 - >32	4 - >32	1 - >32
		KPC-2 (4)	4/4 15 min	>32	>32	>32
		VIM-1 (2)	2/2 120 min	>32	>32	>32
		NDM-1 (2)	2/2	>32	>32	>32
			120 min			
	***E. coli***	OXA-48 (1)	1/1 24 h	16	8	4
		CTX-M-15 (n = 1)	0/2	0.125 - 0.25	0.25 - 0.5	0.064 - 0.125
		CTX-M-15+ CIT (n = 1)	120 min and 24 h			

*peaks m/z: 476.5, 498.5, 520.5 and 542.5 Da.

A synthesis of the results showing the species, resistance mechanism and MIC range in the test panel and validation panel in relation to the results in the hydrolysis assay based on ertapenem.

### Pseudomonas aeruginosa (n = 25)

Six out of elevenVIM producing *P. aeruginosa*, as well as the IMP-14-producing isolate tested, hydrolysed ertapenem after 120 minutes of incubation with the specific ertapenem hydrolysis peak pattern. The hydrolysis was fully inhibited in the presence of DPA in all cases. Ertapenem was not hydrolysed by the non-carbapenemase producing (no carbapenemase confirmed genetically or phenotypically), carbapenem resistant, *P. aeruginosa* isolates (n = 10). Of the 4 *P. aeruginosa* isolates included in the validation panel (three VIM-1 and one VIM-2) were correctly assigned as carbapenemase producers (both VIM-1). The carbapenemase production was inhibited by DPA both for VIM and IMP positive strains. Prolonged incubation (24 h) did not reveal any signs of hydrolysis in the strains tested negative after 120 min incubation (one VIM-1 and one VIM-2). There was no linkage between VIM-type and hydrolysis results. A summary of the results is presented in Table 1.

### Other species (*Acinetobacter baumannii* (n = 4), *Escherichia coli* (n = 3)

None of the 4 *Acinetobacter baumannii* group isolates(OXA-23 like (n = 2), OXA-24-like (n = 1) and OXA-58 like (n = 1)) included in the validation panel hydrolysed ertapenem within 120 min incubation. All isolates, however, displayed the specific pattern of ertapenem hydrolysis after a prolonged incubation (24 h). The two isolates of *E. coli* only producing a classical ESBL-enzyme (two CTX-M-1 group and one CTX-M-1 group plus CIT-group plasmid mediated AmpC) did not hydrolyse ertapenem at any time point. The OXA-48 positive isolate of *E. coli* did not hydrolyse ertapenem within 2 h, but prolonged incubation (24 h) revealed hydrolysis. A summary of the results is presented in Table 1.

## Discussion

The drastic increase of isolates with the ability to produce carbapenemases in Enterobacteriacae, *Acinetobacter* spp. and *P. aeruginosa* rapidly challenges the treatment concept of severely ill patients [1]. Whether the carbapenem resistance is due to carbapenemase production or other mechanisms is considered important for infection control teams. Molecular methods are available for the verification of the genes responsible for carbapenemase production but have the limitation of not detecting new mechanisms [9-11]. The phenotypic assays so far on the market have problems with the time to result, isolates with low expression of the carbapenemase genes and that specific inhibitors are not available for several enzymes [2]. The introduction of MALDI-TOF for the detection of carbapenemases has seemed promising due to the versatile approach of the detection of hydrolysis and the seemingly high sensitivity and specificity [5,7,8]. For the detection of carbapenemases in Acinetobacter the use of imipenem has been chosen [6,8] while for the detection of carbapenemases in Enterobacteriaceae meropenem is best validated but ertapenem has also been suggested [5,7]. Most methods developed so far for this purpose have only investigated very small collections, in all 30 isolates, of *P. aeruginosa*[6,7,12] and only 10 isolates with a VIM enzyme out of which 9 were detected. [6,12]. We included 25 isolates of *P. aeruginosa* out of which 14 carried a VIM enzyme and 1 an IMP-14-enzyme. Only 9 of these isolates could be detected (8 VIM and the only tested IMP positive isolate) using this method based on ertapenem. Both the VIM-type and absence of VIM-production could be ruled out as possible explanations for this. We therefore hypothesize that the non-hydrolysis of ertapenem might be due to additional porin loss resulting in a very low fraction of ertapenem (if any) to reach the periplasmatic site of action of the VIM-enzyme [13]. This finding is important as it shows that the local epidemiological situation where both the mechanism and species of interest may vary is important when choosing the right method for the detection of carbapenemases. However, when a carbapenemase was detected the use of inhibitor could verify the presence of a metallo-β-lactamase also in *P. aeruginosa*.

The rapid verification (45–150 min including the preparation steps, incubation and MALDI-TOF analysis) of carbapenemase production separating KPC isolates from other carbapenemases is to our knowledge the most rapid verification method of carbapenemases in *K. pneumoniae* developed so far. As shown by others, direct detection of carbapenemase production directly from blood culture vials is possible [4] and could be of great importance especially in hospitals with high incidence of carbapenemase producing isolates, as the rescue treatment in these cases is associated with worse patient outcome [14]. We did not have any IMP-producing *K. pneumoniae* isolates available for this study and the specificity for KPC of the 15 min hydrolysis might thus be overrated. However the only IMP-producing *P. aeruginosa* isolate did not hydrolyse ertapenem in 15 min (data not shown).

The method presented here is not dependent on any know-how in molecular biology and could be performed in any laboratory having access to a MALDI-TOF with open software allowing the manual analysis of mass spectra in a m/z range far below the range of 2–20 kDa used for species ID. We choose to build this assay on the hydrolysis of ertapenem as this hydrolysis is associated with specific degradation peaks of 472.5, 494.5, 516.5 and 538.5 easily visualized using the HCCA matrix used for species ID and does not need the addition of SDS (as compared to meropenem) [5]. The method accurately detection of KPCs in *K. pneumoniae* and was not primarily developed for detecting OXA-enzymes but these enzymes were introduced in the “validation panel” to test the robustness of the method in a clinical setting. The method failed to detect OXA-enzymes in the validated time frame of 2 h. However a prolonged incubation for 24 h displayed the hydrolysis pattern in *K. pneumoniae*, *Acinetobacter spp*. and *E.coli* while the controls containing only ertapenem or classical ESBL-producing *E.coli* did not show any signs of spontaneous hydrolysis. Although a bit slow, the method thus seems promising for the detection of the OXA 48-enzyme, but has to be validated further with several more species with varying OXA-enzymes.

The addition of inhibitors, as suggested by others [4,8] in the assay might not be necessary as the time to detection was highly specific for the separation of KPC from MBL-enzymes. However, we did not test isolates positive for IMP-enzymes which might show rapid hydrolysis and if in doubt, both APBA and DPA showed specific inhibition of KPC and MBL enzymes respectively and thus served as further verification of the type of enzyme expressed. In an attempt to streamline the two tests an incubation time of 120 min was tested also for the KPC-verification test. This was however not successful as the high amount of APBA then needed (12 mg/mL) also seemed to inhibit the action of NDM. No hydrolysis could be observed in NDM incubated with high concentration of APBA. The specificity of APBA is thus in this assay dependent on the combination of incubation time and concentration of APBA.

From a methodological point of view the assay was easy to perform and interpret. We used a categorical interpretation of the peaks as being present or not and did not use the intensity ratio between the hydrolysis and non-hydrolysis peaks previously proposed by Sparbier [4]. Similar to Sparbier we observed the peak of 450 Da which is a degradation peak of ertapenem. This peak was by Sparbier observed only when performing a similar assay directly from blood culture [4]. However, in this study the 450-peak was present in all runs but with a higher intensity in the presence of KPC, VIM or NDM. The peak was not included for the interpretation of hydrolysis. For further studies this peak has to be characterized further.

## Conclusions

This method allowed a rapid detection and verification of KPC, NDM and VIM producing *K. pneumoniae* and can be performed at a low cost. This study revealed some caveats regarding the use of this type of hydrolysis assays for the detection of carbapenemases as not all VIM-producing *P. aeruginosa* as well as none of the OXA-48 positive isolates were detected within the 120 min time frame of the assay. Modifications of the assay and/or a change of conditions and carbapenem used might overcome this problem. If the rapid degradation of ertapenem by KPC also with meropenem or imipenem as substrate has to be investigated further and the definite sensitivity and specificity of the assay have to be evaluated on a larger collection of isolates.

## Methods

### Hydrolysis assay

The hydrolysis assay was developed using a Microflex™ (Bruker Daltonics, Billerica, MA, USA) mass spectrometer. The parameters settings were: ion source 1, 19.0 kV; ion source 2, 17.2 kV; lens, 6.0 kV; detector gain, 2.5 kV. Spectra were recorded in the mass range of 0–1000 Da with 60 Hz laser frequency. Each spectrum was obtained from 240 laser shots. The polished steel target plate (Bruker Daltonics, Bremen, Germany) and HCCA matrix (2.5 mg α-cyano-4-hydroxycinnamic acid dissolved in 50% acetonitril, 47.5% HPLC-pure H_2_O and 2.5% trifluoroacetic acid, (Bruker Daltonics)) was used. For calibration the Peptide calibration standard II (Bruker Daltonics) was used. The peaks employed for calibration were CCA [M + H]^+^ at 190.05 Da, CCA [2 M + H]^+^ at 379.09 Da and Bradykinin (1–7) peak [M + H]^+^ at 757.40 Da.

The analysis of MALDI-TOF MS spectra was performed with the Flexanalysis 3.3 software (Bruker Daltonics). The spectra were smoothed and baseline subtracted and then manually examined for the specific ertapenem related peak patterns in the mass range of 4–600 Da previously described [4]. To approve a spectrum as reliable at least one sum buffer peak of hydrolysed or unhydrolysed ertapenem had to have a minimum intensity of 10^4^. The high intensity proves the specificity of the peaks and guarantees that no unspecific background noise is misinterpreted as a significant peak.

### Stability of ertapenem

Ertapenem for intravenous injection (Invanz®, MSD) was used for the hydrolysis assay. 1.0 g of Invanz® was dissolved in 10 ml HPLC-pure water to a concentration of 100 mg/mL. Aliquots of 200 μL were stored at −20°C or +4°C. The stability of ertapenem was tested after one week and 6 months. The ertapenem (100 mg/mL) was thawed and diluted in 10 mM ammonium hydrogen citrate buffer pH 7.1 (ammonium citrate dibasic dissolved in water, Sigma Aldrich) to the concentration 0.5 mg/mL. 2 μL were applied on a polished steel target plate and left to dry and then overlaid with 1uL matrix. A mass spectrum was obtained and a peak pattern consistent with unhydrolysed ertapenem, the presence of the 475.5 Da peak of ertapenem, 498.5 Da [ertapenem + Na]^+^ and 520.5 Da [ertapenem + 2Na]^+^, was considered as conclusive for stability as previously described [4].

### Detection of KPC-, VIM- and NDM-production

Based on the methods described by Sparbier and Hrabak [4,5] an assay for the detection and verification KPC, VIM and NDM production was developed using four isolates of *K. pneumoniae* two isolates with KPC production (CCUG 56233 and a clinical isolate) and two VIM-producing clinical isolates. The assay was based on ertapenem (0.5 mg/mL), a standardized inoculum of 4 McF, an optimal incubation time (15 min KPC and 120 min NDM and VIM) and the determination of the appropriate amount of inhibitor for each incubation time. Inhibitors used were 2,6-Pyridinedicarboxylic acid (DPA) (Sigma Aldrich, Germany; 1.5 mg/mL, dissolved in water,) and 3-aminophenylboronic acid (APBA) (Sigma-Aldrich, Germany; 3.0 mg/mL, dissolved in water) and were freshly prepared for each assay. Peaks generated were manually examined and qualitatively judged by the presence of hydrolysed or unhydrolysed ertapenem respectively.

### Test panel

Seventeen (17) clinical isolates of carbapenemase-producing *Klebsiella pneumoniae* previously classified as KPC- (n = 10, four KPC-2, two KPC-3 and four just verified as KPC), VIM-1 (n = 3) or NDM-1-positive (n = 4) using PCR (9–11) were tested. The carbapenem susceptible *K. pneumoniae* ATCC 13882 and clinical *K. pneumoniae* isolates phenotypically classified as having a classical ESBL (n = 6) or with acquired AmpC, (n = 6) were used as controls. Eleven (11) clinical isolates of carbapenem resistant *Pseudomonas aeruginosa* previously classified as VIM-producing, two VIM-1, six VIM-2, two VIM and one positive for IMP-14, with specific PCR [15,16] were tested together with ten (10) carbapenem resistant clinical isolates phenotypically verified as non-MBL producers. A summary of the tested isolates are presented in Table 1.

All isolates were retrieved on blood agar overnight at 35°C and verified to species using The Microflex™, and the MALDI Biotyper 3.0 software (Bruker Daltonics) using standard parameters. A score value of ≥ 2.0 was considered a reliable species ID. Susceptibility testing was performed for ertapenem, imipenem and meropenem using Etest (BioMérieux, Marcy L´Etoille, France) on Mueller Hinton agar according to the manufacturer’s instructions. Carbapenemase production was verified using the KPC/MBL Confirm ID Kit (Neo-Sensitabs™, Rosco diagnostica A/S) *K. pneumoniae* and for *P. aeruginosa*.

The isolates were analyzed to test the method with the same concentrations as described above. 1.5 mL of a bacterial suspension (4 McF) in 0.9% NaCl was prepared from overnight cultures and centrifuged at 13 400×g during 2 minutes at room temperature. The supernatant was removed by pipetting. The pellet was re-suspended by pipetting in 20 μL of ertapenem (0.5 mg/mL) and incubated for 15 min and 2 h respectively for the detection of hydrolysis. For the verification of carbapenemase production the bacterial pellet was re-suspended in 10 uL ertapenem (1 mg/mL) together with 10 μL APBA (for KPC) or 10 uL DPA (for VIM and NDM). The suspensions were incubated in 35°C for 15 and 120 minutes and then centrifuged at 13 400×g during 2 minutes at room temperature. 2 μL of the supernatant was applied to a polished steel target plate, left to dry, and 1 μL matrix was applied on each spot before analysis with MALDI-TOF MS. For each isolate tested ertapenem alone was incubated 15 or 120 minutes as control of unspecific hydrolysis.

### Validation panel

As a validation set 22 isolates (Table 1) with varying resistance phenotypes and mechanisms were blinded to the primary investigator (ÅJ). The isolates were retrieved on blood agar overnight at 35°C and verified to species ID using The Microflex™, and the MALDI Biotyper 3.0 software (Bruker Daltonics) using standard parameters. All isolates were incubated with ertapenem (0.5 mg/mL) for 15 and 120 min and analyzed according to the final protocol. Isolates positive at any time point were re-incubated together with the inhibitor suitable for the respective time point (i.e. APBA if hydrolysed within 15 min and DPA if hydrolysed within 2 h). Isolates negative in the assay were incubated overnight, as well as ertapenem only as negative control, and analysed after 24 h.

## Competing interests

The authors declare that they have no competing interest.

## Authors’ contributions

ÅJ participated in the design of the study, performed the development of the method and the validation, analysed the data. JE participated in the development of the method and the validation and analysed the data. CGG participated in the study design and the data analysis, and provided strains. MS participated in the design of the study and analysed the data. All authors helped to draft the manuscript. All authors read and approved the final manuscript.
